# Risks of Placenta Previa and Hypertensive Disorders of Pregnancy Are Associated With Endometrial Preparation Methods in Frozen-Thawed Embryo Transfers

**DOI:** 10.3389/fmed.2021.646220

**Published:** 2021-07-22

**Authors:** Yu Tao, Yanping Kuang, Ningling Wang

**Affiliations:** Department of Assisted Reproduction, Shanghai Ninth People's Hospital, Shanghai Jiao Tong University School of Medicine, Shanghai, China

**Keywords:** frozen-thawed embryo transfer, ovarian stimulation, HRT, hormone replacement therapy, hypertensive disorders of pregnancy

## Abstract

**Background:** Endometrial preparation is essential in frozen-thawed embryo transfer (FET) cycles. Recent studies suggested that different endometrial preparation methods may influence obstetrical complications. However, the association between hormone replacement therapy (HRT) and ovarian stimulation (OS) FET endometrial preparation and obstetrical complications remains unknown.

**Methods:** This retrospective cohort study included a total of 79,662 confirmed embryo transfer cycles during the period from January 2003 to December 2019. After exclusion, the remaining cases were categorized into an ovarian stimulation FET group (OS FET group, *n* = 29,121) and a hormone replacement therapy FET group (HRT FET group, *n* = 26,776) and subjected to the analyses. The primary outcome was the rate of obstetrical complications included placenta previa, placenta abruption, hypertensive disorders of pregnancy (HDP), placenta accreta, gestational diabetes mellitus (GDM), preterm premature rupture of the membrane (pPROM). The secondary outcome was pregnancy outcomes such as live birth rate, birth weight, pre-term and post-term delivery and cesarean sections. In order to minimize the bias, 10 pregnancy-related factors were adjusted in multiple logistic regression analysis.

**Results:** Placenta previa (0.6 vs. 1.2%, *P* < 0.001) and HDP (3.5 vs. 5.3%, *P* < 0.001) were found lower in the OS FET than HRT FET group. Cesarean section was observed lower in the OS than HRT group (76.3 vs. 84.3%, *P* < 0.001). After adjustment for 10 important pregnancy-related confounding factors, we found that the risk of placenta previa (aOR 0.54, 95% CI 0.39–0.73) and HDP (aOR 0.65, 95% CI 0.57–0.75) and cesarean section (aOR 0.61, 95% CI 0.57–0.66) were still significantly reduced in the OS than HRT group. Furthermore, live birth rates were higher (80.0 vs. 76.0%, *P* < 0.001), and the miscarriage rate was lower (17.7 vs. 21.3%, *P* < 0.001) for pregnancies conceived with OS FET than with HRT FET. And the average birth weight was lower in the OS group compared to HRT group (2982.3 ± 636.4 vs. 3025.0 ± 659.0, *P* < 0.001), as well as the small-for-gestational age (SGA) was higher (8.7 vs. 7.2%, *P* < 0.001) and the large-for-gestational age (LGA) was lower (7.2 vs. 8.6%, *P* < 0.001) in the OS group than in the HRT group.

**Conclusions:** The risks of placenta previa and HDP were lower in patients conceiving after OS FET than in those after HRT FET. Further prospective studies are required to further clarify the mechanism underlying the association between endometrium preparation and obstetrical complications.

## Introduction

Frozen-thawed embryo transfer (FET) was well recognized to be more efficient by reducing the waste of embryos and repeated oocyte retrieval. Although debates still remains regarding perinatal morbidity (1, 2), evidence has demonstrated less ovarian hyper-stimulation syndrome (OHSS) and comparable live birth rates (LBR) in frozen embryo transfer compared with fresh transfer (3–5), which has resulted in the increased use of frozen cycles (6). Therefore, FET may be a better method of safely treating infertile couples at a relatively low cost.

Different cycle protocols are used for the preparation of the endometrium during FET: natural, artificial and ovarian stimulated cycles. Despite natural cycle, an artificial cycle is a hormone-replacement cycle where endometrium is prepared with exogenous oestrogen followed by progesterone administration before embryo transfer, and in stimulated cycles, the follicular development is induced and controlled via gonadotropins and ovulation is triggered once the ovulation criteria are met. While several studies have investigated the rates of pregnancy, live birth, or miscarriage, the results remain controversial, and the best method of preparing the endometrium for embryo transfer remains unknown (7–9).

Meta-analyses suggested that the risk of hypertensive disorders of pregnancy (HDP) was higher in ART pregnancies than in spontaneously conceived pregnancies (10, 11). Some believed that different preparation methods of the endometrium could effect on extravillous trophoblast (EVT) invasion and vascular remodeling through changes in the decidual cell-derived regulators of hemostasis, fibrinolysis and vascular tone, which might lead to obstetrical complications such as preeclampsia and placenta accrete (12–14). Recent study by Saito et al. showed that patients who conceived with hormone replacement therapy (HRT) FET had increased risks of HDP and placenta accreta and a reduced risk of gestational diabetes mellitus (GDM) in comparison to those who conceived with natural cycle FET (1). However, the associations between HRT FET and ovarian stimulated (OS) FET endometrium preparation and obstetrical complications have hardly been investigated. Hence, the objective of the present study was to clarify the differences of risks of obstetrical complications between patients who conceived after OS FET and after HRT FET.

## Materials and Methods

### Population Study and Design

This retrospective study was carried out at the Reproductive Medicine Centre of the Shanghai Ninth People's Hospital affiliated with the Shanghai Jiao Tong University School of Medicine. These data were collected on a mandatory basis from our EMR (electronic medical record) data base. Every patient was requested to provide data of pregnancy outcomes after embryo transfer in our hospitals and the quality of the data were reassured.

**Figure 1:**
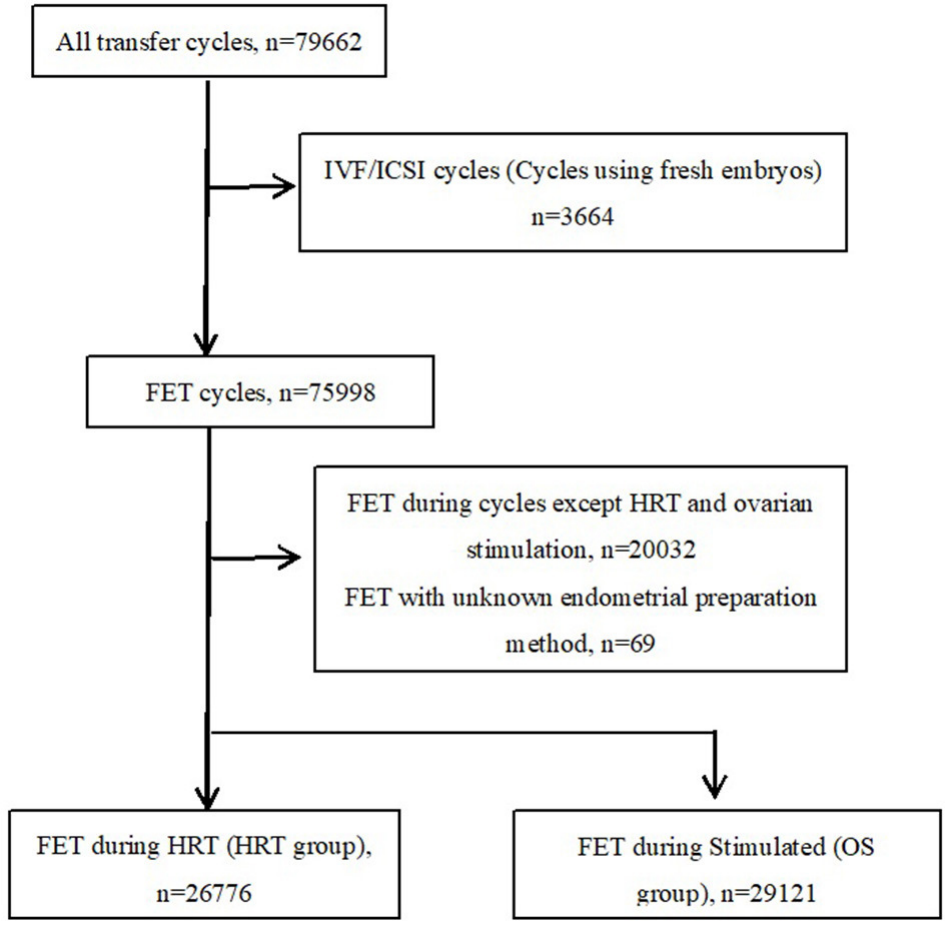
A flow diagram showing the distribution of the study populations.

A total of 79,662 confirmed autologous embryo transfer cycles were recorded in the ART database during the period from January 2003 to December 2019 (Figure 1). We excluded cycles where fresh embryos (*n* = 3,664) were used for transfer. Among the FET cases, cycles except HRT and stimulated cycle (*n* = 20,032) and missing or incomplete data for protocols on the preparation of the endometrium (*n* = 69) were excluded. The remaining cases were categorized into an ovarian stimulation FET group (OS group, *n* = 29,121) and a hormone replacement therapy FET group (HRT group, *n* = 26,776) and subjected to the analyses. In our hospital, the choice of endometrial preparation method is made based on the patients' condition and preference and the discretion of treating physicians. For instance, patients with ovulation disorders often undergo OS or HRT FET, for difficulties with natural ovulation. On the other hand, HRT FET is also chosen due to the convenience of scheduling the date of transfer.

The details of embryo vitrification and thawing methods can be found in our previous article (15). All laboratory procedures remained constant during this research. In brief, cleavage stage embryos were graded with reference to the Cummins criteria (16). Quality assessment of blastocysts was on the basis of the Gardner and School craft scoring system (17). We only thawed embryos on the same day of ET. A maximum of two embryos were allowed to be transferred in all FET cycles.

Endometrial preparation of HRT was performed as described previously (18, 19). In short, oral E2 was commenced on the third day of the menstrual cycle; progesterone exposure was initiated when the endometrial thickness was appropriate (usually ≥7 mm). Embryo transfer was performed 3 days after progesterone administration for day 3 embryos or 5 days later for blastocysts. In OS FET cycles, letrozole was prescribed orally for 5 days initiating on cycle day 3 of menses. Ultrasound monitoring and serum hormone analysis were performed from cycle day 10 onwards. If the leading follicle reached a diameter of ≥14 mm on cycle day 10, transvaginal ultrasound was repeated every 2 days and no other drugs were added until ovulation triggering. In case of a dominant follicle <14 mm on day 10, a daily dosage of 75 IU hMG (Anhui Fengyuan Pharmaceutical Co.) was supplemented to stimulate follicle growth. When the dominant follicle reached a mean diameter of ≥17 mm, the timing of hCG triggering was dependent on the occurrence of an LH surge. On detection of a serum LH surge (LH ≥ 20 IU/l and more than double the average LH level over the past 2 days), a bolus of hCG was injected and luteal phase support (LPS) was started 2 days after trigger. In all study groups, 400 mg daily progesterone vaginal suppositories (Utrogestan; Besins Healthcare) were used during LPS and LPS was continued until 10 weeks of gestation if pregnancy was achieved as previous described (20).

### Follow-Up and Definitions

The primary outcome was the rate of obstetrical complications. Obstetrical complications included placenta previa, placenta abruption, HDP, placenta accreta, GDM, preterm premature rupture of the membrane (pPROM). HDP in this study includes preeclampsia and gestational hypertension and excludes chronic hypertension. GDM is diagnosed based on recommendations by the international association of diabetes and pregnancy study groups (21). As secondary outcomes, we analyzed the rate of live birth, cesarean section, and pre- and post-term delivery and outcomes of the offspring, such as sex and weight at birth. In this study, pregnancy is diagnosed by detecting the gestational sac with vaginal ultrasound. Gestational age was divided into delivery before 37 gestational weeks (preterm delivery), from 37 to 41 weeks (term delivery) and after 41 weeks (post-term delivery). Neonatal birth weight was divided into <2,500 g, between 2,500 and 3,999 g, and ≥4,000 g. In addition, we investigated the neonatal birth weight regarding small- for-gestational age (SGA) and large-for-gestational age (LGA) neonates according to the birth weight reference percentiles for Chinese (22).

### Statistical Analyses

Statistical analyses were carried out using Statistical Package for Social Sciences (SPSS) version 21.0. The numerical data were presented as the mean ± SD, and the categorical variables were shown as % (n/N). Continuous variables were compared with Student's *t*-test. Categorical variables were compared with Pearson's χ^2^ test or with Fisher's exact test when necessary. A *P* value < 0.05 was considered statistically significant.

For all characteristics, we calculated the mean and standard deviation values for continuous variables and the number of the cases for discrete variables for both the OS and HRT groups. The pregnancy outcomes such as live birth, miscarriage, and stillbirth rates were calculated using the number of successful pregnancies as the denominator. The obstetrical outcomes including complications and cesarean delivery were calculated using the number of live births as the denominator. The outcomes of the offspring such as sex and weight at birth were calculated using the number of neonates as the denominator. The crude and adjusted odds ratios (OR and aOR) of OS FET compared with HRT FET for pregnancy outcomes and obstetrical complications were evaluated by multiple logistic regression analysis. In order to minimize the bias, we adjusted for the following 10 potential confounders: maternal age at embryos transfer, maternal BMI, infertility duration, maternal smoking history, gravidity, parity, cause of infertility, number of embryos transferred, methods of fertilization and embryo developmental stage at transfer. Unadjusted and adjusted odds ratios (ORs) and 95% confidence intervals (CIs) were calculated by the regression models.

## Results

### Baseline Characteristics of OS FET and HRT FET Groups

Table 1 shows the characteristics of two groups of the study populations. In the OS FET group, the average maternal age was lower, the gravity and parity were lower, number of transferred embryos was higher, and IVF fertilization method was less often used than in the HRT FET group. Maternal BMI and smoking history were comparable between the two groups. And there were also no differences between the two groups regarding the cause of infertility, infertility duration and embryo developmental stage at cryopreservation.

**Table 1:** Baseline characteristics of OS FET and HRT FET groups.

**Characteristics**	**OS FET group (*n* = 29,121)**	**HRT FET group(*n* = 26,776)**	***P*-value**
Maternal age at transfer (years)	32.41 ± 4.69	34.02 ± 5.41	<0.001
Maternal BMI (kg/m^2^)	21.99 ± 3.08	21.96 ± 3.06	0.210
Infertility duration (years)	3.47 ± 2.88	3.51 ± 3.12	0.150
Maternal smoking history	116 (0.4%)	89 (0.3%)	0.223
Gravity			<0.001
0	15,792 (54.2%)	12,610 (47.1%)	
1	6,837 (23.5%)	6,441 (24.1%)	
≥2	6,492 (22.3%)	7,725 (28.8%)	
Parity			<0.001
0	26,185 (89.9%)	23,411 (87.4%)	
≥1	2,936 (10.1%)	3,365 (12.6%)	
Cause of infertility			0.296
Female factor	17,880 (61.4%)	16,279 (60.8%)	
Male factor	3,611 (12.4%)	3,347 (12.5%)	
Mixed	5,649 (19.4%)	5,355 (20.0%)	
Number of embryo transferred			<0.001
1	5,650 (19.4%)	6,161 (23.0%)	
2	23,471 (80.6%)	20,615 (77.0%)	
Embryo fertilization methods			<0.001
IVF	17,242 (59.2%)	16,850 (62.9%)	
ICSI	8,315 (28.6%)	7,484 (28.0%)	
IVF+ICSI	3,564 (12.2%)	2,442 (9.1%)	
Developmental stage at cryopreservation			0.208
Cleavage stage (day 3)	24,885 (85.5%)	22,780 (85.1%)	
Blastocyst (day 5)	4,236 (14.5%)	39,96 (14.9%)	

*Data are presented as mean ± SD for continuous variables and n/n (%) for dichotomous variables. All P values were assessed with the use of χ2 or Student's t test. HRT, hormone replacement therapy; OS, ovarian stimulation; FET, Frozen embryo transfer*.

### Pregnancy Outcomes of OS FET and HRT FET Groups

The outcome of pregnancy and obstetrical and offspring's outcomes of live birth cases were shown in Table 2. Among pregnancies, live birth rates were higher (80.0 vs. 76.0%, *P* < 0.001), and the miscarriage rate was lower (17.7 vs. 21.3%, *P* < 0.001) for pregnancies conceived with OS FET than those conceived with HRT FET. The rates of stillbirth and ectopic pregnancy were comparable between the two groups. Induced abortion due to congenital malformation was lower in OS than in HRT group (0.7 vs. 1.0%, *P* = 0.023). Both pre- and post-term births were comparable between the two groups. Cesarean section was observed less frequently among pregnancies due to OS FET than among those due to HRT FET (76.3 vs. 84.3%, *P* < 0.001). Twin births was found more frequently among pregnancies due to OS FET than HRT FET (26.3 vs. 22.6%, *P* < 0.001). Placenta previa (0.6 vs. 1.2%, *P* < 0.001) and HDP (3.5 vs. 5.3%, *P* < 0.001) were found less reported in OS FET than HRT FET group. However, Placenta abruption, placenta accreta, GDM and pPROM were comparable between the two groups.

**Table 2:** Pregnancy outcomes of OS FET and HRT FET groups.

**Characteristics**	**OS FET group**	**HRT FET group**	***P*-value**
**Outcome of pregnancy**	**(*****n*** **=** **14,504)**	**(*****n*** **=** **11,183)**	
Live birth	11,599 (80.0%)	8,497 (76.0%)	<0.001
Still birth	5 (0.1%)	4 (0.1%)	0.778
miscarriage	2,573 (17.7%)	2,382 (21.3%)	<0.001
Induced abortion due tocongenital malformation	106 (0.7%)	112 (1.0%)	0.023
ectopic pregnancy	221 (1.5%)	188 (1.7%)	0.343
**Obstetrical outcomes of live birth cases**	**(*****n*** **=** **11,599)**	**(*****n*** **=** **8,497)**	
Gestational age at birth, weeks	37.65 ± 2.04	37.69 ± 2.12	0.161
Gestational age category			0.052
≤36 weeks	2,106 (18.1%)	1,615 (19.0%)	
37–41 weeks	9,487 (81.8%)	6,871 (80.9%)	
≥42 weeks	6 (0.1%)	11 (0.1%)	
Mode of delivery			<0.001
Vaginal delivery	2,746 (23.7%)	1,338 (15.7%)	
Cesarean section	8,853 (76.3%)	7,159 (84.3%)	
Number of offspring			<0.001
Singleton Twin	8,550 (73.7%) 3,049 (26.3%)	6,580 (77.4%) 1,917 (22.6%)	
Obstetrical complications			
Placenta previa	69 (0.6%)	103 (1.2%)	<0.001
Placenta abruption	22 (0.2%)	12 (0.1%)	0.515
HDP	411 (3.5%)	450 (5.3%)	<0.001
Placenta accreta	8 (0.1%)	11 (0.1%)	0.252
GDM	1,016 (8.8%)	715 (8.4%)	0.404
pPROM	242 (2.1%)	160 (1.9%)	0.334
**Outcomes of the offspring in live birth cases**	**(*****n*** **=** **14,648)**	**(*****n*** **=** **10,414)**	
Sex of offspring			
Male	7,614 (52.0%)	5,425 (52.1%)	0.859
Female	7,034 (48.0%)	4,989 (47.9%)	
Birth weight, g	2982.3 ± 636.4	3025.0 ± 659.0	<0.001
Birth weight category			
<2,500g	2,881 (19.6%)	1,994 (19.2%)	<0.001
2,500–3,999g	11,100 (75.8%)	7782(74.7%)	
≥4,000g	667 (4.6%)	638 (6.1%)	
Small for gestational age (SGA)	1,272 (8.7%)	755 (7.2%)	<0.001
Large for gestational age (LGA)	1,058 (7.2%)	900 (8.6%)	<0.001

*Data are presented as mean ± SD for continuous variables and n/n (%) for dichotomous variables. All P values were assessed with the use of χ2 or Student's t test. HDP, hypertensive disorders of pregnancy; GDM, gestational diabetes mellitus; pPROM, preterm premature rupture of the membrane; other abbreviations see in Table 1*.

Regarding the offspring's outcome, the average birth weight was lower in OS FET group compared to HRT FET group (2982.3 ± 636.4 vs. 3,025.0 ± 659.0, *P* < 0.001). The proportions of infants with birth weight <2,500 g was higher (19.6 vs. 19.2%, *P* < 0.001) and ≥4,000 g was lower (4.6 vs. 6.1%, *P* < 0.001) in OS FET group, as well as rates of SGA were higher (8.7 vs. 7.2%, *P* < 0.001) and LGA were lower (7.2 vs. 8.6%, *P* < 0.001) in OS FET group than HRT FET group. However, the sex of offspring was comparable between the two groups.

### Logistic Regression of Obstetrical Outcomes of OS-FET vs. HRT-FET

Through the logistic regression model, in crude analyses, we found that the risk of cesarean section (OR 0.60, 95% CI 0.56–0.65, *P* < 0.001), placenta previa (OR 0.49, 95% CI 0.36–0.66, *P* < 0.001) and HDP (OR 0.66, 95% CI 0.57–0.75, *P* < 0.001) were significantly reduced in OS FET group than in HRT group (Table 3).

**Table 3:** Logistic regression of obstetrical outcomes of OS-FET vs. HRT-FET.

**Outcomes**	**Unadjusted OR (95% CI)**	***P*-value**	**Adjusted OR (95% CI)**	***P*-value**
Cesarean section	0.60	<0.001	0.61	<0.001
	(0.56–0.65)		(0.57–0.66)	
Preterm delivery	0.95 (0.88–1.02)	0.125	0.91 (0.85–0.98)	0.012
Post-term delivery	0.39 (0.15–1.08)	0.071	0.36 (0.13–0.98)	0.046
**Obstetrical complications**				
Placenta previa	0.49 (0.36–0.66)	<0.001	0.54 (0.39–0.73)	<0.001
Placenta abruption	1.34 (0.67–2.72)	0.411	1.31 (0.64–2.66)	0.459
HDP	0.66 (0.57–0.75)	<0.001	0.65 (0.57–0.75)	<0.001
Placenta accrete GDM	0.53 (0.21–1.32) 1.05 (0.95–1.16)	0.175 0.390	0.552 (0.22–1.38) 1.09 (0.98–1.21)	0.205 0.090
pPROM	1.11 (0.91–1.36)	0.309	1.06 (0.87–1.30)	0.552

*Multivariable logistic regression analyses was performed and analyses were adjusted for maternal age at embryos transfer, maternal BMI, infertility duration, maternal smoking history, gravidity, parity, cause of infertility, number of embryos transferred, methods of fertilization and embryo developmental stage at transfer. OR, odds ratio; CI, confidence interval; other abbreviations see in Tables 1, 2*.

After adjustment for 10 important pregnancy-related confounding factors, such as maternal age at embryos transfer, maternal BMI, infertility duration, maternal smoking history, gravidity, parity, cause of infertility, number of embryos transferred, methods of fertilization and embryo developmental stage at transfer, we found that the risk of cesarean section (aOR 0.61, 95% CI 0.57–0.66, *P* < 0.001), placenta previa (aOR 0.54, 95% CI 0.39–0.73, *P* < 0.001) and HDP (aOR 0.65, 95% CI 0.57–0.75, *P* < 0.001) were still significantly reduced in OS FET group than in HRT group. Furthermore, the risk of pre-term delivery (aOR 0.91, 95% CI 0.85–0.98, *P* = 0.012) and post-term delivery (aOR 0.36, 95% CI 0.13–0.98, *P* = 0.046) was also reduced in OS FET than in HRT group after correcting for confounders (Table 3).

## Discussion

In our large retrospective cohort study, we demonstrated for the first time that OS FET is significantly associated with less risks of HDP and placenta previa compared with HRT FET. We also showed that the pregnancies conceived with HRT FET had an increased incidence of cesarean and pre and post-term delivery.

Previous studies have reported an increased incidence of HDP and placenta accreta among pregnancies after FET when compared to fresh embryo transfer (9, 23). Roumundstad et al., studied the Medical Birth Registry of Norway between 1988 and 2002, and concluded that there was a six-fold higher risk of placenta previa in singleton pregnancies and a three-fold higher risk of placenta previa in twins conceived by ART compared with naturally conceived pregnancies (24). Suzuki and Kato reported that velamentous umbilical cord insertion was independently associated with *in vitro* fertilization use (OR 4.82, 95% CI 3.3–7.1) (25). To our knowledge, the specific mechanisms underlying this difference remain unknown. In our study, we demonstrated an association between HRT endometrium preparation method and elevated risks of HDP and placenta previa which suggested that different endometrial preparation methods might really influence the later development of obstetrical complications. Known risk factors of HDP are multiple pregnancies (26). However, in our study, the multiple pregnancies rate was lower in HRT FET group than that in OS FET group, yet the adjusted OR is still significantly higher in HRT FET (Table 3). Another known risk factor of HDP was maternal age, including young or advanced maternal age (26), in our study, the average maternal age in OS FET group was lower than in HRT FET group, however after adjusted for maternal age and other pregnancy-related cofounders, HRT was still the risk factor of HDP while undergoing FET. Placenta accreta is a serious complication which might lead to serious perinatal obstetric outcomes even maternal death and the surgical histories of uterus and placenta previa are risk factors for placenta accrete (27). Although the rate of placenta previa was found higher in HRT FET group in our study, the frequency of placenta accrete was comparable between the two groups.

As is well known, HRT requires medication with supra-physiologic hormones (7). However, based on the current literature it is not possible to identify the optimum hormonal levels and the optimum duration of HRT in FET endometrium preparation (7). During early human pregnancy, extravillous trophoblast (EVT) cells from the placenta invade the uterine decidual spiral arterioles and mediate the remodeling of these vessels, and estradiol and progesterone may control EVT movement and induces decidualization of the endometrial stromal cells. Aberrant hormone levels in early pregnancy may cause defects in EVT invasion which can manifest as the serious pregnancy complication such as pre-eclampsia (13).

In a prospective cohort study of 260 Caucasian women, they concluded that an increased serum progesterone level in the early third trimester (27th week) has a role in the development of pre-eclampsia featuring superficial placentation (RR = 2.65, 95% CI, 1.46–4.81) (28). On the other hand, a decreased progesterone level in the early pregnancy might also lead to abnormal placentation such as placenta accretes through failure of well decidualization and over invasion of EVT (29, 30). A more recent study showed that the risks of HDP and placenta accreta were higher in patients conceived with HRT FET than in those conceived with natural cycle FET (1). And a study even suggested that different type of progesterone in LPS may associate with altered risk of pre-eclampsia (31). In our study we confirmed that the risks of HDP were higher in HRT FET than OS FET, indicating that HRT FET might associate with the highest risk of HDP, suggesting that HRT FET women might need closer monitoring than those women undergo with other endometrium preparation methods.

Previous study also found that the risk of GDM after HRT FET was significantly lower than natural cycle FET (1). To our knowledge, GDM is associated with maternal characteristics, such as age, obesity, and ethnicity (32). It was reported that the risk of GDM is two-fold higher in women with singleton pregnancies conceived following ART compared with women who conceived spontaneously, and progesterone use during pregnancy might be an important risk factor to the development of gestational diabetes (33). The diabetogenic effects of progesterone in pregnancy were mainly explained by the enhancement of insulin resistance by the hormone, especially in skeletal muscle and adipose tissue, through a reduction in glucose transporter 4 (GLUT4) expression (34). More convincingly, progesterone receptor-knockout mice were found to have improved glucose tolerance (35). Saito et al. believed that the decreased secretion of insulin-counteracting hormones from the placenta might suppress the pathogenesis of GDM in some HRT-FET-derived pregnancies, hence decreased the GDM rate in HRT FET pregnancies compared to natural cycles (1). However, in our study, we found that the rate of GDM were comparable between the HRT FET and OS FET groups.

In this study, we found a significant higher rate of miscarriage and a significant lower live birth rate among pregnancies after HRT FET than after OS FET. While previous studies exploring the optimum endometrium preparation for FET obtained the same results showing that pregnancy loss rate was significantly lower for OS than for HRT FET cycles, and the live birth rate was significantly higher for OS than for HRT FET cycles (36). Study by Zong et al. found that after adjusting for the pregnancy-related factors such as age, BMI, antral follicle count (AFC), et al., HRT FET were at an increased risk of low birth weight (LBW) compared to natural cycle group (37). And study by Saito et al. showed that risks of pre-term birth and post-term birth were elevated in the HRT FET group compared with natural cycle group (1). In our study, when compared to HRT FET, we found that the average birth weight was lower, and the rate of SGA was higher and LGA was lower in OS FET. However, when we analyzed birth weight of singleton and twin births separately, we found the birth weight was comparable between the two groups (Supplementary Table 1). This differs from a study by Ishii et al., claiming that the average birth weight from HRT FET was significantly greater than that of an ovulatory cycle FET (38); however, an ovulatory cycle FET also involves natural cycle FET. HRT FET reduces the need for repeated hospital visits and enables patients with or without ovulation disorders to schedule FET at their leisure (7), and currently it is the most popular endometrium preparation method worldwide. Nevertheless, we should also consider obstetrical risks when we decide on the endometrium preparation method, since OS FET and HRT FET patients harbor different risks for obstetrical complications, as we found in our study.

This study has its own strength. Firstly, it has a large sample size. In order to investigate relatively infrequent events, like minor obstetrical complications, a large sample size is essential. In this sense, our sample size was large enough to assess the risk of various obstetrical complications. Secondly, it is the first study compared OS to HRT in FET endometrium preparation focusing on obstetrical complications. This study is a retrospective cohort study, with inherent associated bias. With this in mind, we adjusted 10 important pregnancy-related confounding factors, such as maternal age at embryos transfer, maternal BMI, infertility duration, maternal smoking history, gravidity, parity, cause of infertility, number of embryos transferred, methods of fertilization and embryo developmental stage at transfer in our analyses in order to minimize possible flaws in our data, and all of these efforts were missing from existing studies. We have to mention that, our database were not perfect, for example, family and drug taking history and associated risk factors including dilation and curettage (D&C), scarred uterus due to adenomyosis or fibroid surgery cannot be obtained from our EMR database. Nevertheless, our results might merit clinicians' attention in clinical practices by closer monitoring patients undergo with HRT endometrium preparation methods.

In conclusion, this is the first study to demonstrate associations between the endometrium preparation methods OS vs. HRT and obstetrical complications. We included more than seventy thousand FET cycles data and found that the risks of HDP and placenta previa were lower in patients conceiving after OS FET than in those conceiving after HRT FET. Further prospective studies are required to confirm and to clarify the mechanism underlying the association between endometrium preparation and obstetrical complications.

## Data Availability Statement

The original contributions presented in the study are included in the article/Supplementary Material, further inquiries can be directed to the corresponding author/s.

## Ethics Statement

The studies involving human participants were reviewed and approved by Ethic Committee of Shanghai Ninth People's Hospital. The patients/participants provided their written informed consent to participate in this study.

## Author Contributions

NW supervised the entire study, including the procedures, conception, design and completion, participated in the interpretation of the study data, and in revisions to the article. YK were responsible for the collection of data. YT contributed the data analysis and drafted the article. All authors contributed to the article and approved the submitted version.

## Conflict of Interest

The authors declare that the research was conducted in the absence of any commercial or financial relationships that could be construed as a potential conflict of interest.
