# Supplementary material for: Risks of Placenta Previa and Hypertensive Disorders of Pregnancy Are Associated With Endometrial Preparation Methods in Frozen-Thawed Embryo Transfers
Source: Front Med (Lausanne). 2021 Jul 22;8:646220. doi: 10.3389/fmed.2021.646220 (PMC8339408; doi:10.3389/fmed.2021.646220)
Supplement: Supplementary file 1 [file Table_1.DOCX]

**Supplementary table 1.**

**Birth weight of singleton and twins between OS FET and HRT FET groups**

| **Characteristics** | **OS FET group** | **HRT FET group** | **P-value** |
| --- | --- | --- | --- |
| Singleton | (n=8550) | (n=6580) |  |
| Birth weight, g | 3321.3±509.7 | 3334.2±541.0 | 0.131 |
| Birth weight category |  |  | 0.749 |
| ＜2500g | 384 (4.5%) | 360 (5.5%) |  |
| 2500-3999g | 7505 (87.8%) | 5587 (84.9%) |  |
| ≥4000g | 661 (7.7%) | 633 (9.6%) |  |
| Twins | (n=6098) | (n=3834) |  |
| Birth weight, g | 2507.1±470.9 | 2494.3±481.3 | 0.191 |
| Birth weight category |  |  | 0.227 |
| ＜2500g | 2497 (40.9%) | 1634 (42.6%) |  |
| 2500-3999g | 3595 (59.0%) | 2195 (57.3%) |  |
| ≥4000g | 6 (0.1%) | 5 (0.1%) |  |

Note: Data are presented as mean±SD for continuous variables and n/n (%) for dichotomous variables. All P values were assessed with the use of χ2 or Student’s t test. HRT=hormone replacement therapy; OS=ovarian stimulation; FET=Frozen embryo transfer.
